# Efficacy and Safety of Tripterygium Wilfordii Hook F on Psoriasis Vulgaris: A Systematic Review and Meta-Analysis of Randomized Controlled Trials

**DOI:** 10.1155/2018/2623085

**Published:** 2018-04-22

**Authors:** Meng Lv, Jingwen Deng, Nan Tang, Yuejin Zeng, Chuanjian Lu

**Affiliations:** ^1^Department of Dermatology, Guangdong Provincial Hospital of Chinese Medicine, Guangzhou 510120, China; ^2^Psoriasis Clinical and Basic Research Team, Guangdong Provincial Academy of Chinese Medical Sciences, Guangzhou 510120, China; ^3^Department of Dermatology, Guangzhou Red Cross Hospital, Guangzhou 510000, China; ^4^Guangdong Provincial Key Laboratory of Clinical Research on Traditional Chinese Medicine Syndrome, Guangzhou 510120, China

## Abstract

**Background:**

Psoriasis is a chronic autoimmune-mediated skin disease that is characterized by persistent localized erythematous scaly plaque. Tripterygium wilfordii Hook F (TwHF), a well-known Chinese medicine that has been used for centuries in China to treat immune diseases, inflammation, and tumor, is accompanied by a degree of toxic effects. Its clinical efficacy and safety on psoriasis are incompletely understood.

**Aim:**

To summarize evidence concerning the efficacy and safety of TwHF in treating psoriasis*. Methods*. EMBASE, Ovid MEDLINE, PubMed, Web of Science, Springer, Cochrane Library, CNKI, CBM, Wanfang, and VIP database were searched up to October 2017. The included literature was assessed and extracted by two independent reviewers. To enhance the available evidence, a systematic review was performed to examine all relevant published literature relating to randomized controlled trials (RCTs) of TwHF. Relative ratios (RRs) and 95% confidence intervals (CIs) were calculated, and a meta-analysis was conducted with RevMan 5.3 software.

**Results:**

Twenty eligible RCTs with 1872 participants were included for systematic review and meta-analysis. Studies were assessed using the Cochrane risk of bias tool. The meta-analysis of add-on effect of TwHF conferred benefit for psoriasis: combination treatment with compound glycyrrhizin (four studies, OR = 0.34, 95% CI 0.22–0.52, *P* < 0.00001, *I*^2^ = 0%), combination treatment with acitretin (three studies, OR = 0.25, 95% CI 0.10–0.63, *P* = 0.003, *I*^2^ = 50%), and combination treatment with compound amino-polypeptide tablet (three studies, OR = 0.37, 95% CI 0.22–0.63, *P* = 0.0002, *I*^2^ = 0%).

**Conclusions:**

Despite several mild side effects of TwHF, there is evidence that TwHF is an effective therapy for psoriasis. However, the conclusions are limited by the small number of included trials. More well-designed RCTs with extensive follow-up periods are warranted to clarify the effects and safety of TwHF in treating psoriasis.

**Trial Registration:**

This review was registered in the International Prospective Register of Systematic Reviews (CRD42016041363).

## 1. Introduction

Psoriasis vulgaris is an immune-abnormal chronic skin disease characterized by well-delineated red, scaly plaques. Psoriasis affects approximately 2%-3% of the population worldwide, and it can significantly impair a participant's wellbeing and physical and mental functioning [1]. Convention therapies include nonspecific immunosuppressants, such as topical steroids and cyclophosphamide, and more specific compounds, such as cyclosporine or other drugs thought to act as immunomodulators (fumarates and intravenous immunoglobulins). There is increasing evidence for immunomodulatory and partly immunosuppressive mechanism of action of tripterygium wilfordii Hook F (TwHF), which has been widely used in China to treat various autoimmune and inflammatory diseases, including psoriasis, rheumatoid arthritis (RA), systemic lupus erythematosus (SLE), ankylosing spondylitis, and idiopathic IgA nephropathy [2]. Thus, compounds such as triptolide or triptolide derivatives may have the potential to be developed as a new class of drugs for immune diseases [3]. Currently, in clinical settings, the preparations of tripterygium wilfordii Radix are in the form of tripterygium, tripterygium total terpenoids, tripterygium glycosides, and tripterygium bilayer tablets, as well as triptolide ointment, which has great application value [2]. Tripterygium glycosides can be extracted as full root extract tripterygium tablets. Tripterygium tablets are used more to treat rheumatoid arthritis in the early stage, and tripterygium glycosides have the widest application. At present, tripterygium preparations are commonly used to treat various kidney disease, thyroid disease, rheumatism, and connective tissue disease.

In recent years, this medicine also has demonstrated applications in dermatology. The treatment of psoriasis with TwHF is well-accepted by clinicians; however, it lacks scientifically reliable evidence. This study provides a systematic review of all relevant published literature relating to randomized controlled trials (RCTs) of TwHF to further clarify the effect and safety of the TwHF treatment in psoriasis.

## 2. Methods

This review included all RCTs using TwHF on treatments of psoriasis. We compared TwHF either alone or in combination against a placebo or another active intervention. The primary outcome of this meta-analysis was the remission of psoriasis lesion.

### 2.1. Literature Search and Selection

A systematic search of articles was conducted using the following electronic databases: EMBASE, Ovid MEDLINE, PubMed, Web of Science, Springer, Cochrane Library, CNKI, CBM-Database, Wanfang database, and VIP database. Reference lists and citations of the identified studies were also screened for relevant publications and to investigate the efficacy and safety of TwHF in treating psoriasis.

### 2.2. Eligibility Criteria

Studies were included if they were prospective RCTs that compared orally administered TwHF (either alone or in combination with other active interventions) with placebo or active medicine.

### 2.3. Exclusion Criteria

Studies were excluded if they met the following exclusion criteria:

1. Participants had comorbidities.
2. The publications lacked original data for the meta-analysis and review articles.
3. The data did not predominantly concern vulgaris type psoriasis (e.g., Guttate, pustular, erythrodermic, inverse, and nail psoriasis).
4. TwHF was used externally.

### 2.4. Study Selection

In the first stage of the study selection process, the titles and abstracts of the papers searched by keywords were examined to exclude irrelevant articles. Next, the full texts of all selected studies were screened according to the inclusion and exclusion criteria.

### 2.5. Data Extraction and Quality Assessment

Using a data extraction form developed in advance, two reviewers (Lv and Deng) independently extracted the following information: first author, publication year, sample size, participant age, intervention, duration, and outcome. The main outcome was the reduction of Psoriasis Area and Severity Index (PASI) (Table 1). Adverse events were also recorded (Table 2). Two reviewers (Lv and Deng) independently conducted risk of bias assessments using the Cochrane Collaboration tool for assessing risk of bias.

### 2.6. Statistical Analysis

Meta-analysis was performed in RevMan (Version 5.3. Copenhagen: The Nordic Cochrane Centre, The Cochrane Collaboration, 2014). Risk ratios (RR) with 95% confidence intervals (CI) for dichotomous data and mean differences (MD) with 95% CIs for continuous data were reported. For studies on a similar type of intervention, we conducted meta-analysis using a fixed-effect model to calculate a pooled intervention effect estimate across trials when the *I*^2^ statistic was less than 50% with reasonable clinical homogeneity. If the *I*^2^ statistic was 50% to 80%, we applied a random-effects model [4]. Meta-analysis was performed according to the different interventions, such as TwHF alone or in combination. And different combinations (compound glycyrrhizin (CG), acitretin, and compound amino-polypeptide tablet (CAPT)) were considered as different interventions.

## 3. Results

### 3.1. Literature Search

**Figure 1:**
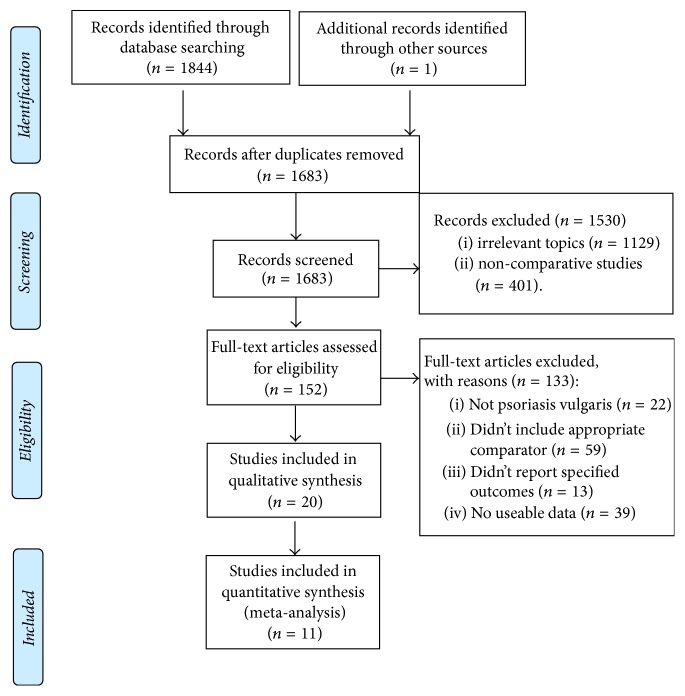
A PRISMA flowchart of the study selection process.

After removing duplicates, we identified 1845 references from our electronic searches (Figure 1). 1683 articles remained after duplicates were removed. After scanning titles and abstracts, we discarded 1530 articles because of irrelevant topics or noncomparative studies. Next, by full-text articles assessing, 136 articles were excluded because they were not for psoriasis vulgaris; did not include appropriate comparator; did not report specified outcomes; or are without useable data. Among the remaining 20 publications for the systematic review, only 11 publications included quantitative synthesis meta-analyses.

### 3.2. Description of Studies

The 20 included RCTs, involving 1872 participants with psoriasis vulgaris, were conducted in hospitals in China and published in Chinese from 1999 to October 2017. Characteristics of the included studies are summarized in Table 1. One study [5] was a randomized, double-blind, double-dummy, parallel-group clinical trial. And only this mentioned the details of random sequence generation and allocation concealment. Three studies [5–7] designed the blinding of participants and outcome assessment. The sample sizes for the included studies ranged from 39 to 200, and the participant age interval ranged from 16 to 78 years.

### 3.3. Description of Interventions

Among the included studies, placebo was used as control only in one study [7]. Four studies [5, 6, 8, 9] reported TwHF as monotherapy compared active interventions (cyclosporin A, acitretin, CG, and Qingdai capsule). There were five studies [10–14] that included the comparison of TwHF plus CG and CG. Three studies were compared TwHF alone or plus acitretin with acitretin [15–17]. In three studies [18–20], TwHF plus CAPT was compared with CAPT alone. Other studies [21–24] combined TwHF with conventional therapies: tazarotene, NB-UVB, and traditional Chinese medicine.

### 3.4. Outcome Measurements

The reduction of disease severity was assessed based on the change in the lesion reduction.

PASI score was used in clinical trials to measure outcomes following psoriasis treatment. The PASI measures the redness, thickness, and scaling of the lesions and the area of involvement, with a total score ranging from 0 to 72. PASI-60, referring to a 60% reduction in PASI, was considered to be effective in treating psoriasis treatment. Twelve of the twenty studies [8–10, 12, 13, 15–20, 22] reported PASI-60 as primary outcome, which was consistent with the Consensus of Diagnosis and Treatment of Psoriasis Vulgaris with Integrative Medicine [25]. One study [5] reported PASI-75 and three studies [6, 7, 24] reported PASI-70 as primary outcome.

According to the disease of TCM syndrome diagnosis curative standard, skin lesions that reduced 60% or above were also accepted as being effective. Other three studies [14, 21, 23] used skin lesions reduction as the effectiveness indicators.

Of all studies, two studies reported the relapse rates at the end of the follow-up period [6, 24], but the definitions of relapse were not stated.

### 3.5. Dropouts and Withdrawals

Wu et al. 2015 [5] reported that there was a higher attrition rate in the acitretin group (5.2% in the TwHF group and 7.0% in the acitretin group by week 2, and 8.6% in the TwHF group and 12.3% in the acitretin group by week 8. Discontinuation occurred because of withdrawn consent (*n* = 1), loss to follow-up (*n* = 8), adverse event (AE) (*n* = 1), and disease progression (*n* = 2). The reasons for discontinuing the study were similar between two groups. He 2008 [17] reported two withdrawals because of AEs in the acitretin group, one because of high cholesterol and one because of serious cheilitis. Zhang et al. 1999 [7] reported that two people withdrew in the TwHF group and that they had gastrointestinal-related adverse reactions. No dropouts were reported in the other studies.

### 3.6. Risk of Bias Assessment

Only two studies [5, 15] stated the details of random sequence generation and one study [5] clarified allocation concealment, and others remained unclear. Attempts were made to contact the original authors to obtain further information about randomization and allocation on the unclear studies. However, the authors of the studies could not be reached.

For “sequence generation,” two studies [5, 15] stated that they used random number tables generated by computer which were assessed as “low risk”; the others were assessed as “unclear.” And beside Wu et al. 2015, the allocation concealment of other studies was not clearly defined. Therefore, the other nineteen studies were assessed as “unclear” in “allocation concealment” because of the lack of information. Three studies that used double-dummy methods or placebo [5–7] were assessed as “low risk” in “blinding,” and the other studies were assessed as “high risk” because of different types of dosage forms or combinations with other types of medicines. Three study [5, 7, 17] had withdrawals in the trial, and they were assessed as “high risk” for “incomplete outcome data.” And the other studies were assessed as “unclear.” All studies were assessed as “low risk” for “selective reporting” because they reported the outcomes prespecified in the methods. All studies were assessed as “low risk” for “other bias,” which refers to the inappropriate influence of cofounders.

**Figure 2:**
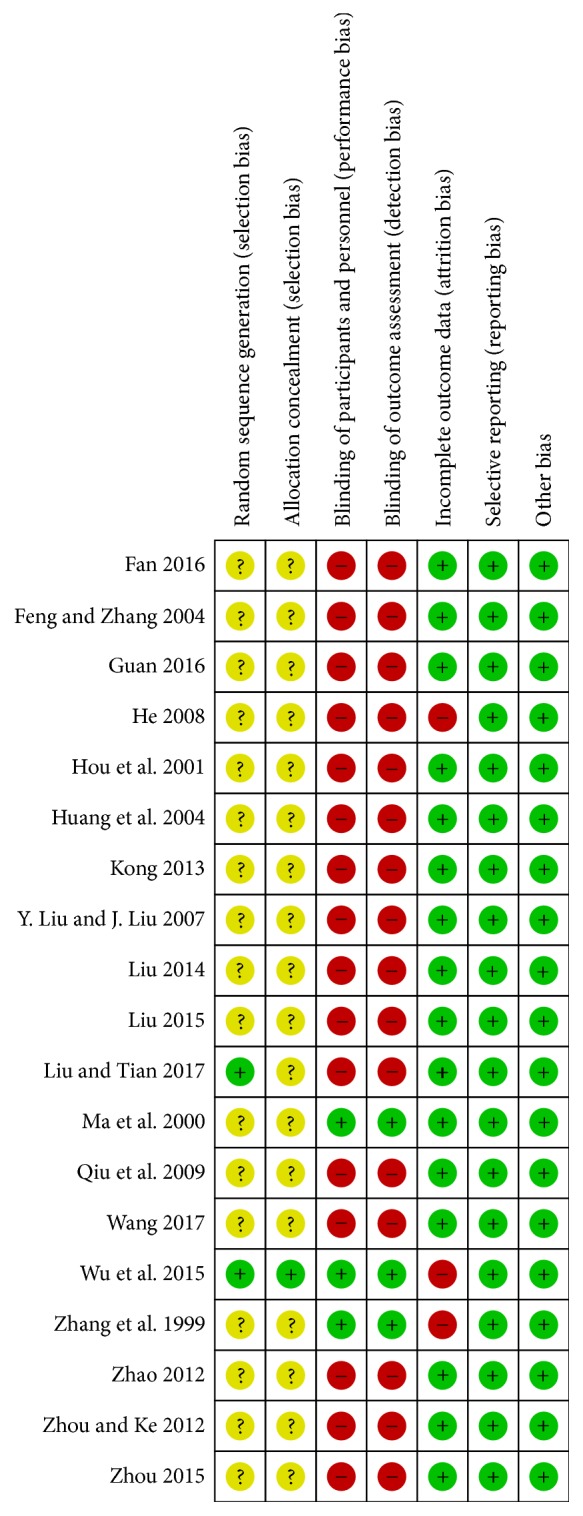
Risk of bias summary.

Risk of bias assessments is presented in Figure 2.

### 3.7. Effects of Interventions

#### 3.7.1. TwHF Alone for Psoriasis Vulgaris

Only one study [19] compared TwHF with placebo. Results of the study showed that there was significant clinical improvement in participants treated with TwHF. No severe side effects of TwHF were observed. Four studies [5, 6, 8, 9] compared TwHF with other active interventions. Liu 2015 [8] showed that tripterygium glycosides could improve psoriasis by inhibiting the activation of T lymphocytes, and its effect and safety were better than cyclosporine A. Wu et al. 2015 [5] conducted a randomized, double-blind, double-dummy, parallel-group clinical trial to prove that there was no significant difference in treatment efficacy between the TwHF and acitretin, but there were fewer treatment-related adverse events in the TwHF group. In Qi et al. 2009 [9], it was equivalence effect of TwHF and glycyrrhizin. In Ma et al. 2000 [6], comparing with Qingdai capsules, tripterygium wilfordii had a fast effect on advanced psoriasis, but long-term follow-up was needed for its curative effect.

#### 3.7.2. Add-On Effect on TwHF for Psoriasis Vulgaris

**Figure 3:**
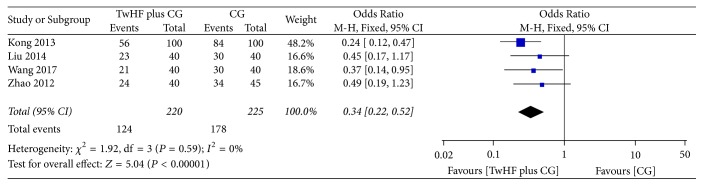
Effect of TwHF plus CG for psoriasis vulgaris.

*(1) TwHF plus CG for Psoriasis Vulgaris*. The main component of CG, glycyrrhizic acid, a triterpenoid saponin glycoside, consists of one molecule of 18 *β*-glycyrrhetic acid as aglycon and two molecules of glucuronic acid, which is known as the most efficacious composition of licorice [26, 27]. Glycyrrhizic acid has been proved to have antiallergic, antiviral, and anti-inflammatory activities [28, 29]. There were four studies [10–14] which included the comparison of TwHF plus CG and CG alone. The results showed that the participants in combination therapy group got more benefit than in the GC group (Figure 3, four studies, OR = 0.34, 95% CI 0.22–0.52, *P* < 0.00001, *I*^2^ = 0%; *P* = 0.02, *I*^2^ = 11%; *P* < 0.00001, *I*^2^ = 0%).

**Figure 4:**
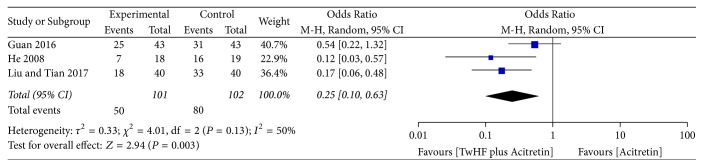
Effect of TwHF plus acitretin for psoriasis vulgaris.

*(2) TwHF plus Acitretin for Psoriasis Vulgaris*. Acitretin is the pharmacologically active metabolite of etretinate, which is highly efficacious as monotherapy in psoriasis and currently approved by the FDA. It has dose-sparing effects when used as combination therapy with conventional systemic drugs. It is a good option for long-term maintenance therapy [30]. When TwHF plus acitretin was compared with acitretin alone in three studies [15–17], the pooled result of PASI-60 was statistically significant (Figure 4, three studies, OR = 0.25, 95% CI 0.10–0.63, *P* = 0.003, *I*^2^ = 50%).

**Figure 5:**
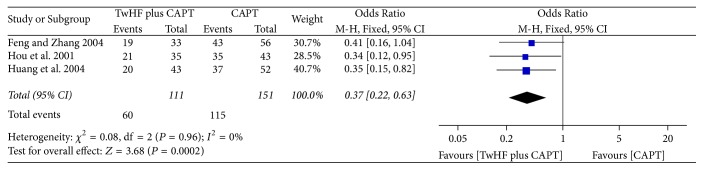
Effect of TwHF plus CAPT for psoriasis vulgaris.

*(3) TwHF plus CAPT for Psoriasis Vulgaris.* The main component of CAPT, aminopeptidase, is an active substance extracted from animal organs. It contains a variety of amino acids, peptides, and trace elements, which helps to regulate the immune function of the body and is conducive to the body's nutritional metabolism. In three studies [18–20], TwHF plus CAPT was compared with CAPT alone. The pooled result of PASI-60 was statistically significant (Figure 5, three studies, OR = 0.37, 95% CI 0.22–0.63, *P* = 0.0002, *I*^2^ = 0%).

### 3.8. Adverse Events

Fourteen studies reported AEs (Table 2). Four studies did not mention any information about AE [14, 18, 21, 23]. Two studies reported that there were no AEs in the two groups [10, 13]. In the TwHF group, the frequent complaints were menstrual disorders in females, dry mouth, gastrointestinal complaints, swelling of the lower limbs, abnormal hepatocytes, and abnormal routine blood results; several studies clearly reported the numbers of different AEs, while some only recorded the AEs and did not provide accurate numbers [5, 6]. Two participants in the control group (acitretin group) withdrew because of AEs, one had high cholesterol, and one had serious cheilitis [17]. Another study reported that one participant in the acitretin group withdrew because of AEs [5]. Two participants withdrew in the TwHF group, both of them because of gastrointestinal adverse reactions [7]. Most of the AEs returned to normal without additional treatment by reducing or stopping the dosage of intervention. However, the relationships between the AEs and the interventions were not further discussed in any study. No severe AEs were reported.

## 4. Discussion

Psoriasis vulgaris is a chronic immune-mediated cutaneous inflammatory disease. TwHF is often used to treat a variety of immune and inflammation-related diseases, such as psoriasis and RA in China due to their favorable cost benefit ratio. The extracts of TwHF contain more than 70 ingredients; however, triptolide is the most potent bioactive substance [31, 32]. It has been shown to possess potent anti-inflammatory and immunosuppressive properties in vitro, as well as in different animal models in numerous preclinical studies [33]. Numerous preclinical studies have demonstrated that the extracts from the root of TwHF inhibit the expression of proinflammatory cytokines, proinflammatory mediators, adhesion molecules, and matrix metalloproteinases by macrophages, lymphocytes, synovial fibroblasts, and chondrocytes. TwHF also induces apoptosis in lymphocytes and synovial fibroblasts and inhibits their proliferation [2]. The major side effects of TwHF reported in the literature are skin dry and rash, gastrointestinal complaints, decreases in erythrocytes and leukocytes, and reproductive toxicity such as dysmenorrhea, irregular menstruation, or reversible sterility. However, serious adverse events have rarely been reported [33].

From the meta-analysis, tripterygium wilfordii Hook F might be an effective and safe treatment in participants with psoriasis vulgaris. In this review, we focused on the efficacy and safety of TwHF. Five studies [5–9] reported TwHF as monotherapy compared placebo or active interventions. Some medicines (cyclosporin A, acitretin, CG, and Qingdai capsule) used as control were regarded as an effective drug treatment for psoriasis. We did not pool data for these five studies because the medicines used in control group were not comparable. However, these studies indicated TwHF was effective in psoriasis vulgaris. Differing from the results of TwHF used as monotherapy, when TwHF combined with other medicines, such as CG, acitretin, and CAPT, the quantitatively assessment showed that the effect of intervention groups was better than that of the control groups.

More than one in seventh patients who were taking TwHF had experienced AEs. Menstrual disorders in females, dry mouth, and gastrointestinal complaints were the most commonly reported AEs following oral TwHF; 14 people had menstrual disorders in 7 studies, but none of them withdrew from the studies. Compared with acitretin, TwHF treatment might have a lower incidence of AE. Gastrointestinal reactions were reported in the two groups. Two participants in TwHF group withdrew from the studies because of serious gastrointestinal reactions, but both of them did not need other treatment. Some participants were unnormal in physical examination such as liver function and routine blood test. All participants returned to normal after reducing the dosage of the medicine or stopping it completely. TwHF should not be used for prolonged periods of time because it will increase risk of adverse reproductive outcomes and its enteron irritation.

Although TwHF was effective in treating psoriasis, the design of many of the studies, which have been published and discussed, may not meet the state-of-the-art criteria for clinical trials today. In addition, significant improvement of PASI in participants with only mild forms of psoriasis may bias a favorable efficacy outcome. Another drawback in better understanding the benefit-risk profile of triptolide is the lack of long-term trials or even reports of open studies lasting for more than one year. Based on published data to date, it is difficult to judge the full potential of this drug for use in psoriasis. Most of the studies did not clearly mention “the sequence generation” and “blinding”; therefore, most of them were low quality studies. Moreover, the sample sizes of the included studies were not sufficiently large.

## 5. Conclusions

This meta-analysis indicated that tripterygium wilfordii Hook F might be an effective and safe treatment in participants with psoriasis. Nevertheless, despite our rigorous methodology, the inherent limitations of the included studies prevent us from reaching a definitive conclusion. Future large-volume, well-designed RCTs with extensive follow-up periods are warranted to confirm and update the findings of this analysis.

## Figures and Tables

**Table 1:** Description of studies.

Publication	Sample size (intervention : control)	Age (mean (SD) or range) (intervention : control)	Intervention	Control	Duration	Outcome	Results
Liu and Tian 2017 [15]	40 : 40	-	Tripterygium glycosides 20 mg tid; acitretin capsules 20 mg once daily	Acitretin capsules 20 mg once daily	8 weeks	PASI 60	82.5%; 45%; *P* < 0.05

Wang 2017 [10]	40 : 40	39.28 ± 4.51; 39. 98 ± 5.03	Tripterygium glycosides 20 mg tid; compound glycyrrhizin 50 mg tid	Compound glycyrrhizin 50 mg tid	8 weeks	PASI 60	77.5%; 52.5%; *P* < 0.05

Fan 2016 [21]	98 : 98	30.4 ± 6.5 : 31.2 ± 6.8	Tripterygium glycosides 20 mg tid; 0.05% tazarotene gel once daily	0.05% tazarotene gel once daily	3 months	75% reduction rate	33.67%: 33.69%: *P* > 0.05

Guan 2016 [16]	43 : 43	36.9 ± 7.6 : 36.8 ± 7.8	Tripterygium glycosides 10 mg tid; acitretin capsules 30 mg once daily	Acitretin capsules 30 mg once daily	8 weeks	PASI 60	72.1%: 58.1%; *P* < 0.05

Liu 2015 [8]	55; 55	31.2 ± 6.4; 32.1 ± 5.8	Tripterygium glycosides 20 mg tid;	Cyclosporin A 2.5 mg/kg/day; after 4 weeks, increase dose to 3–5 mg/kg/day	12 weeks	PASI 60	63.6%; 47.3%; *P* < 0.05

Wu 2015 [5]	58 : 57	42.0 ± 12.0 : 40.2 ± 12.4	Tripterygium glycosides 20 mg tid; placebo matching acitretin 30 mg once daily	Acitretin 30 mg once daily; placebo matching TwHF 20 mg tid	8 weeks	PASI 75	19.0%; 17.5%; *P* > 0.05

Zhou 2015 [11]	34 : 33	36.2 ± 4.7 35.9 ± 5.3	Tripterygium glycosides 20 mg tid; compound glycyrrhizin 400 mL injection once daily	Compound glycyrrhizin 400 mL injection once daily	2 months	PASI 30	97.1%; 81.8%; *P* < 0.05

Liu 2014 [12]	40 : 40	-	Tripterygium glycosides 20 mg tid; compound glycyrrhizin 60 mL injection once daily	Compound glycyrrhizin 60 mL injection once daily	4 weeks	PASI 60	75%; 57.5%; *P* < 0.05

Kong 2013 [13]	100 : 100	32.78 ± 18.21 : 31.5 ± 17.23	Tripterygium glycosides 20 mg tid; compound glycyrrhizin 60 mL injection once daily	Compound glycyrrhizin 60 mL injection once daily	4 weeks	PASI 60	84%; 56%; *P* < 0.05

Zhao 2012 [14]	45 : 40	36.5 ± 4.4 : 35.5 ± 5.1	Tripterygium glycosides 20 mg tid; compound glycyrrhizin 40 mL injection once daily	Compound glycyrrhizin 40 mL injection once daily	8 weeks	30% reduction rate	75.6%; 60.0%; *P* < 0.05

Zhou and Ke 2012 [22]	60 : 60	36.58 ± 4.92 : 37.12 ± 4.32	Tripterygium glycosides 20 mg tid for first 4 weeks and 10 mg tid for last 4 weeks; NB-UVB 3 times per week	NB-UVB 3 times per week	8 weeks	PASI 60	88.33%; 63.33%; *P* < 0.05

Qi et al. 2009 [9]	48 : 47	-	TwHF 20 mg tid;	Compound glycyrrhizin 60 mL injection once daily	8 weeks	PASI 60	81.25%; 76.60%; *P* < 0.05

Qiu et al. 2009 [23]	52 : 50	-	Tripterygium glycosides 20 mg tid Chinese medicine Fuling decoction once daily	Compound Qingdai pills 6 g tid	3 months	60% reduction rate	90.4%; 56.0% *P* < 0.05

He 2008 [17]	19 : 20	34.3 ± 10.1 : 35.5 ± 14.3	Tripterygium glycosides 20 mg tid; acitretin capsules 0.5–1 mg/kg·d bid or tid	Acitretin capsules 0.5–1 mg/kg·d bid or tid	8 weeks	PASI 60	84.2%; 38.9%; *P* < 0.01

Y. Liu and J. Liu 2007 [24]	36 : 34	33.5 : 35.8	Tripterygium glycosides 10–20 mg tid; Chinese medicine Xiaoyin decoction once daily	Compound Qingdai pills 6 g*∗*3 day^−1^	4 weeks	PASI 70	86.1%; 64.7% *P* < 0.05

Feng 2004 [18]	56 : 33	-	Tripterygium glycosides 20 mg tid; compound amino-polypeptide Tablets 5 pieces bid	Compound amino-polypeptide tablets 5 pieces bid	10 weeks	PASI 60	76.78%; 57.57% *P* > 0.05

Huang 2004 [19]	52 : 43	36.7 ± 18.2 : 34.7 ± 19.3	Tripterygium glycosides 10 mg tid; Compound Amino-polypeptide tablets 5 pieces bid	Compound amino-polypeptide tablets 5 pieces bid	60 days	PASI 60	71.15%; 46.51% *P* < 0.01

Hou 2001 [20]	43 : 35	31.4 : 33.2	Tripterygium glycosides 20 mg tid + compound amino-polypeptide tablets 5 pieces bid	Compound amino-polypeptide tablets 5 pieces bid	30 days	PASI 60	81.4%; 60.0%; *P* < 0.05

Ma 2000 [6]	50 : 50	28 ± 2.3 : 29.54 ± 13.22	Tripterygium glycosides capsules 6 g tid	Qingdai capsules 6 g tid	4 weeks	PASI 70	84%; 84%; *P* > 0.05

Zhang 1999 [7]	25 : 27	26.68 ± 12.3 : 28.62 ± 14.23	Tripterygium glycosides capsules 6 g tid	Placebo capsules 6 g tid	4 weeks	PASI 70	84% 11.11% *P* < 0.01

**Table 2:** Adverse events reported in the studies.

Publication	Adverse events (number)		AE rate (%)	
	Intervention group	Control group	Intervention group	Control group
Liu and Tian 2017 [15]	Mouth dryness and anorexia (1)	Gastrointestinal reactions (2)	2.5%	2.5%.
Wang 2017 [10]	No AEs	No AEs	0%	0%
Fan 2016 [21]	Not mentioned	Not mentioned	-	-
Guan 2016 [16]	Gastrointestinal reactions (2), mouth or skin dryness (11)	Gastrointestinal reactions (3) Mouth or skin dryness (11)	32.6%	32.2%
Liu 2015 [8]	Anorexia (1), diarrhea (2), menstrual disorders in females (2)	Serum creatinine and urea nitrogen increased (4); Anorexia (2) Diarrhea (1)	9.1%	12.7%
Wu et al. 2015 [5]	Menstrual disorders in females, dry mouth, gastrointestinal reactions, and swelling of the lower limbs (undefined)	Dry mucosa, facial pigmentation, hair loss, paronychia, palpitation (undefined)	43.6%	78.1%
Zhou 2015 [11]	Anorexia (1), diarrhea (2), menstrual disorders in females (2)	Serum creatinine and urea nitrogen increased (4); Anorexia (2) Diarrhea (1)	9.1%	12.7%
Liu 2014 [12]	Nausea (1)	Anorexia (1); Serum creatinine increased (1)	2.9%	6.1%
Kong 2013 [13]	Not AEs	Not AEs	0%	0%
Zhao 2012 [14]	Not mentioned	Not mentioned	-	-
Zhou and Ke 2012 [22]	Skin dry or pain (2), gastrointestinal reactions (8), hepatocytes abnormal (1), routine blood abnormal (2)	Skin dry or pain (4)	21.67%	6.7%
Qi et al. 2009 [9]	Serum creatinine increased (2)	Not AEs	4.26%	0%
Qiu 2009 [23]	Not mentioned	Not mentioned	-	-
He 2008 [17]	Menstrual disorders in females (2), routine blood abnormal (1)	Dry mucosa or skin or eyes (12), hair loss (1)	15.79%	65%
Y. Liu and J. Liu 2007 [24]	Gastrointestinal reactions (1)	Not AEs	2.78%	0%
Feng and Zhang 2004 [18]	Not mentioned	Not mentioned	-	-
Huang et al. 2004 [19]	Skin dry or pain (14), gastrointestinal reactions (3), hepatocytes abnormal (1), routine blood abnormal (2)	Skin dry or pain (12).	38.46%	27.91%
Hou et al. 2001 [20]	Mouth or skin dry, gastrointestinal reactions (29)	Mouth dryness (23)	67.44%	65.71%
Ma et al. 2000 [6]	Menstrual disorders in females (1), gastrointestinal reactions (2)	Gastrointestinal reactions (undefined)	6%	-
Zhang et al. 1999 [7]	Menstrual disorders in females (1), gastrointestinal reactions (6)	Not AEs	28%	0%
